# First-in-human study of anticancer immunotherapy drug candidate mobilan: safety, pharmacokinetics and pharmacodynamics in prostate cancer patients

**DOI:** 10.18632/oncotarget.27549

**Published:** 2020-04-07

**Authors:** Natalia V. Eremina, Vasily I. Kazey, Sergey V. Mishugin, Roman V. Leonenkov, Dmitry Y. Pushkar, Vadim L. Mett, Andrei V. Gudkov

**Affiliations:** ^1^Panacela Labs LLC, Moscow, Russian Federation; ^2^D.D. Pletnev Municipal Clinical Hospital, Moscow Department of Healthcare, Moscow, Russian Federation; ^3^St. Petersburg Clinical Research and Practical Center for Specialized Oncological Medical Care, St. Petersburg, Russian Federation; ^4^S.I. Spasokukotsky Municipal Clinical Hospital, Moscow Department of Healthcare, Moscow, Russian Federation; ^5^Buffalo BioLabs LLC, Buffalo, NY, USA; ^6^Department of Cell Stress Biology, Roswell Park Comprehensive Cancer Center, Buffalo, NY, USA

**Keywords:** prostate cancer, immunotherapy, mobilan, adenoviral vector, intratumor injection

## Abstract

Toll-like receptor 5 (TLR5) controls endogenous immune responses to pathogens and is a promising target for pharmacological stimulation of anti-tumor immunity. Mobilan is an innovative gene therapy agent consisting of a non-replicating bicistronic adenovirus directing constitutive expression of human Toll-like receptor 5 (TLR5) and the secreted flagellin-based TLR5 agonist, 502s. In mice, Mobilan injection into prostate tumors resulted in autocrine TLR5 signaling, immune system activation, and suppression of tumor growth and metastasis. Here we report a first-in-human placebo-controlled clinical study of Mobilan aimed at evaluating safety, tolerability, pharmacokinetics and pharmacodynamics of a single intra-prostate injection of Mobilan in early stage prostate cancer patients. Mobilan was safe and well-tolerated at all tested doses; thus, the maximum tolerated dose was not identified. Injection of Mobilan induced signs of self-resolving inflammation not present in placebo-injected patients, including transient elevation of PSA and cytokine (G-CSF, IL-6) levels, and increased lymphoid infiltration in prostate tissue. The highest dose of Mobilan (10^11^ viral particles) produced the best combination of safety and pharmacodynamic effects. Therefore, Mobilan is well-tolerated and induces the expected pharmacodynamic response in humans. These results support further clinical development of Mobilan as a novel immunotherapy for prostate cancer.

## INTRODUCTION

Prostate cancer (PC) is the fourth most common type of malignant neoplasm in the world, being surpassed only by breast, lung and colorectal cancers. In men, only lung cancer is more prevalent. 1.3 million new PC diagnoses were made worldwide in 2018. In the United States (US), an annual incidence rate of ~170,000 makes PC the most common of all cancers in males (excluding non-melanoma skin cancers) [1]. Prostate cancer is also the most common cancer among males in European countries, with a prevalence of 214 cases per 100,000 males [4]. In addition to its high incidence, PC also accounts for a large proportion of all cancer-related deaths. PC ranks second among oncological diseases with respect to mortality in males worldwide [2, 3].

Approaches for treating localized PC include conventional methods such as delayed treatment (expectant management or active surveillance), radical prostatectomy (RPE), radical radiotherapy, and hormone therapy, as well as a number of experimental methods (cryoablation, focal therapy, high-intensity focused ultrasound therapy, etc.) [5, 6].

Gene therapy-mediated immunotherapy is a promising novel approach to treatment of many types of cancer, including prostate cancer [7, 8]. In general, immunotherapeutic strategies are designed to combat cancer either by externally stimulating the immune system to modulate its response to tumor cells or by inducing presentation of exogenous tumor-specific antigens to the immune system. These tumor-specific exogenous antigens may be artificial or natural and are designed to be recognized by the immune system.

Data obtained over the past several decades confirm that targeted modification of immune responses can lead to destruction of tumor cells and improve the survival rate of cancer patients. Currently, three gene therapy drugs are registered and used in oncological practice – Gendicine™ and Oncorine™ in China and Imlygic^®^ in the USA [9–11].

As key regulators of immune responses, Toll-like receptors (TLRs) have strong potential as targets for various strategies aimed at prevention and treatment of cancer. TLRs (1-13) recognize conserved pathogen-associated molecular patterns that are expressed by a broad range of microorganisms and, upon ligand binding, initiate signaling pathways leading to activation of innate and adaptive immune responses. In particular, TLR5 recognizes the bacterial flagellin protein and is expressed on the surface on a variety of immune cells, including monocytes, macrophages, neutrophils, lymphocytes, NK cells, and dendritic cells. Ligand-activated TLR5 signaling ligand initiates a MyD88-dependent cascade causing activation of NF-κB and subsequent upregulation of multiple cytokines, and type 1 interferons and is inhibited via proapoptotic pathways [12]. Activation of TLRs enhances the capacity of dendritic cells to capture antigens from the environment, which are then presented to T cells upon interaction between the innate and adaptive immunity systems.

**Figure 1:**
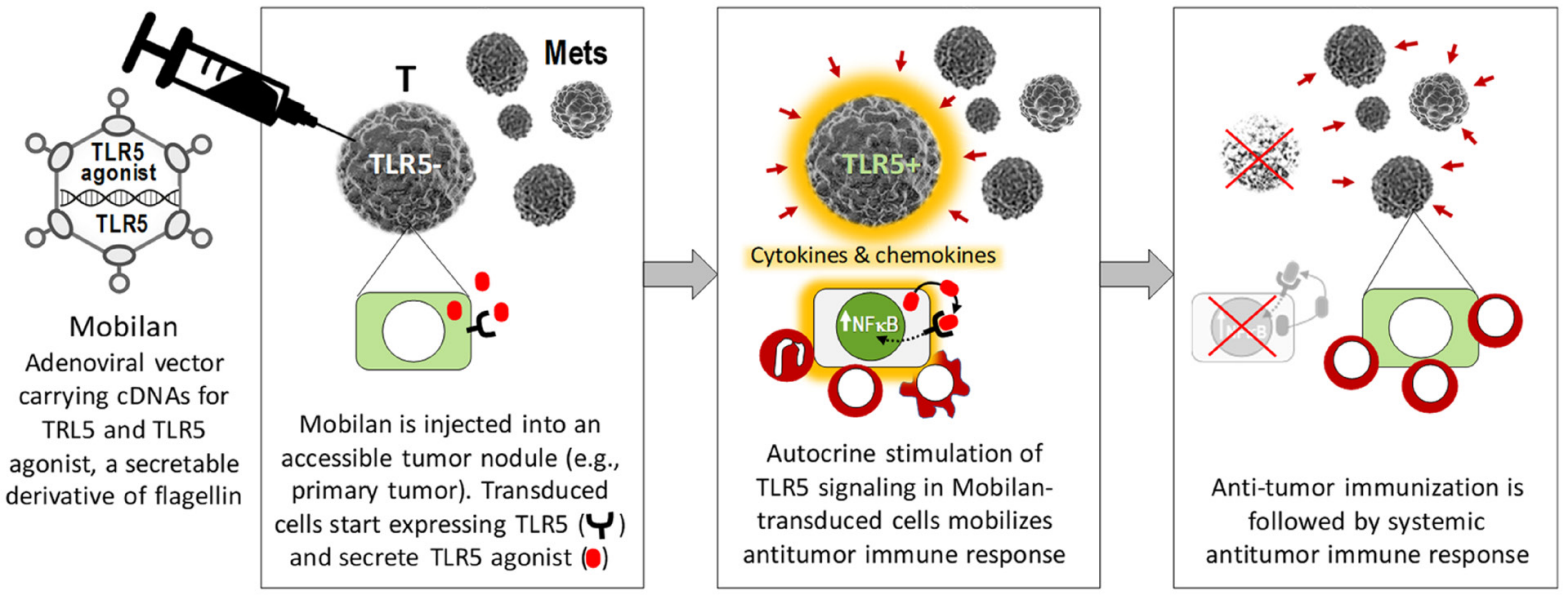
Schematic illustration of the mechanism of action of Mobilan resulting in activation of antitumor immune responses.

Mobilan (M–VM3) is an innovative gene therapy agent that consists of recombinant non-replicating bicistronic adenovirus that directs expression of human Toll-like receptor 5 (hTLR5) and 502s, a secreted form of a pharmacologically optimized flagellin derivative that acts as a selective agonist of TLR5 (Figure 1). Infection of tumor cells with Mobilan results in constitutive autocrine/paracrine stimulation of the TLR5 signaling pathway, which leads to induction of an innate immune response followed by development of an adaptive antitumor immune response. This proprietary technology makes it possible to convert any tumor node that is accessible for injection and suitable for adenovirus infection (i.e., has cell surface expression of coxsackievirus and adenovirus receptor) into an “*in situ* vaccine” capable of activating and recruiting therapeutic immune responses [13].

The program of preclinical studies of antitumor drug Mobilan involved studies that aimed to select the optimal therapeutic area, to confirm the relevance of the mechanism of drug action with respect to the selected therapeutic area, and to study its efficacy using an animal model. Tumor types for which the adenovirus-based delivery system will be effective were selected by conducting a number of screening studies. In these studies, the tumors have successfully been classified and the tumor types most susceptible to transduction by agents based on the adenoviral vector gene delivery system (e. g., Mobilan) were revealed using objective criteria (presence of the coxsackievirus and adenovirus receptor (CAR) on the cell surface). Preliminary studies aimed at selecting a therapeutic target revealed that the majority of prostate tumors are CAR-positive and can be transduced by the genes contained in the drug with the adenovirus gene delivery system. Hence, immunotherapy of prostate cancer upon intratumor/intraprostatic administration is one of the optimal areas of Mobilan application [13].

Here we report the results of the first-in-human trial of Mobilan. This trial was designed to evaluate the safety and tolerability of a single intra-tumoral injection of different doses of Mobilan in patients diagnosed with prostate cancer (stages T1–T2, M0, N0). In addition, the pharmacokinetics of this immunotherapeutic agent, its pharmacodynamic effects on cytokines, immune cells, and PSA levels, and its impact on prostate tumor tissue structure were evaluated.

## RESULTS

### Study design and procedures

The trial was a single-blind, randomized, placebo-controlled phase I study aimed at evaluating the safety, tolerability, pharmacokinetics, and pharmacodynamics of a single intra-prostatic injection of Mobilan in patients diagnosed with prostate cancer, with dose escalation. The primary objective of the study was to evaluate safety and tolerability of the drug candidate, ideally leading to identification of the maximum tolerated dose (MTD). Secondary objectives were to evaluate associated pharmacokinetic and pharmacodynamic variables including those indicative of drug efficacy.

Patients were recruited for the trial and treated at three clinical sites in the Russian Federation. Thirty-four patients ranging in age from 53 to 74 years were screened for participation in the study. Thirty-two patients were included in the study: thirty randomized patients had prostate cancer of clinical stage T2 according to the TNM classification, and two patients had prostate cancer of stage T1. Study subjects were randomized in accordance with the previously developed randomization scheme into five cohorts for Mobilan treatment at different dose levels cohorts or placebo treatment as shown in Table 1. Within each cohort, patients were assigned to Mobilan or placebo treatment at a 3:1 ratio. Thus, overall 24 patients received Mobilan, referred to herein as the investigational product (IP), and 8 received placebo.

**Table 1:** Disposition of patients in study cohorts

Cohort	Dose of Mobilan (M-VM3), particles	Number of patients receiving Mobilan	Number of patients receiving placebo
1	1 × 10^9^	9	3
2	3 × 10^9^	3	1
3	1 × 10^10^	3	1
4	3 × 10^10^	3	1
5	1 × 10^11^	6	2

The dose of IP administered to study subjects was increased three-fold from cohort to cohort beginning with the no-effect level dose of 1 × 10^9^ viral particles (as determined in preclinical studies [unpublished data]) in Cohort 1. Accordingly, dose levels were 1 × 10^9^, 3 × 10^9^, 1 × 10^10^, 3 × 10^10^ and 1 × 10^11^ viral particles for Cohorts 1 through 5, respectively. The IP was administered as a single transrectal injection into both prostate lobes of each patient using transrectal ultrasonography as a guide. The total injected volume of IP (in 5% glucose solution) per patient was 1 ml, with equal distribution between prostate lobes and at different depths in each lobe (3–5 injection sites/depths of 100–150 μl for each lobe). Placebo-treated patients were injected with 5% glucose solution delivered in an identical manner as the IP.

**Figure 2:**
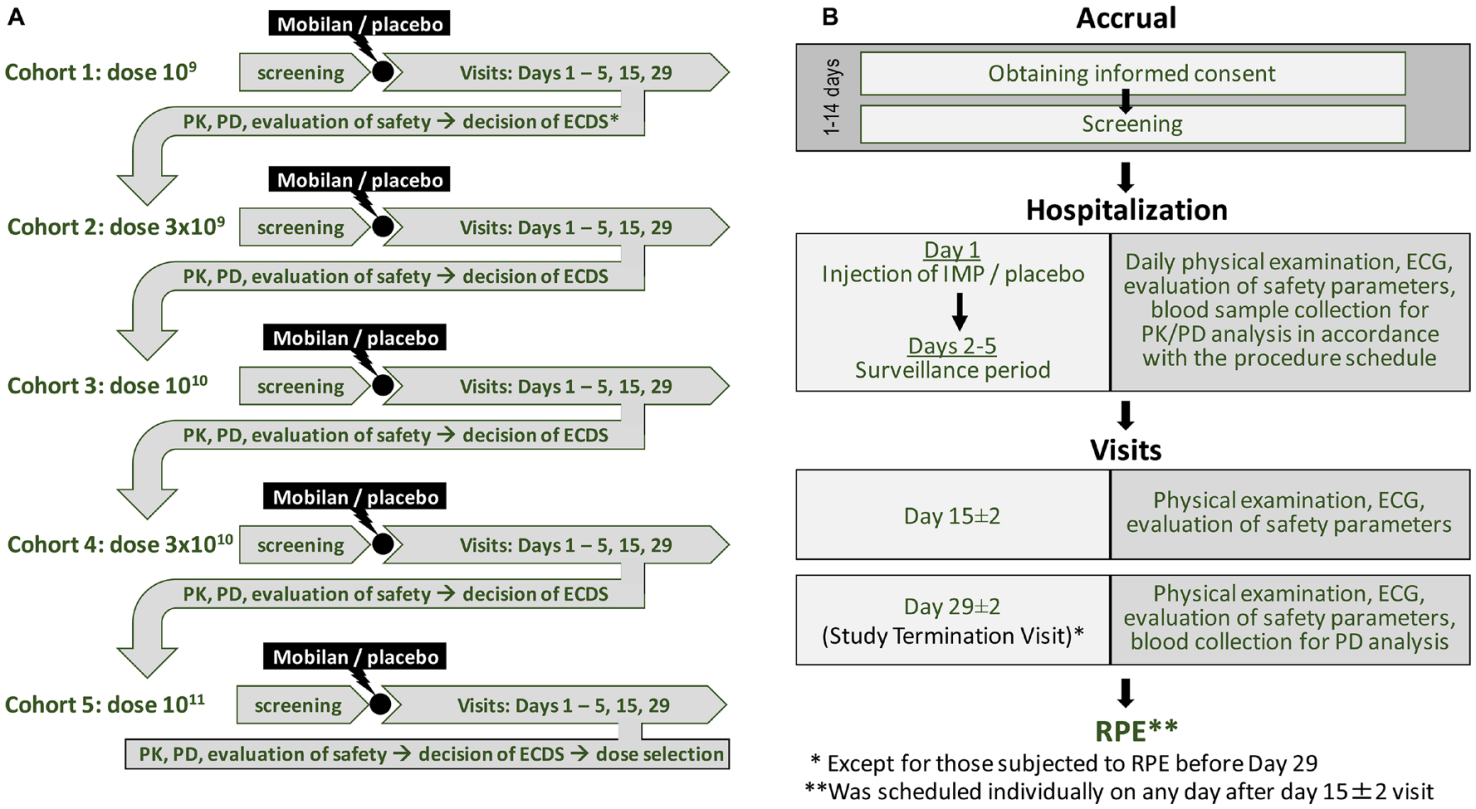
Study design for the first-in-human Phase I clinical trial of Mobilan (**A**) and schedule of study stages (**B**). See text for details.

The study design is illustrated graphically in Figure 2. The trial consisted of several periods: Screening, Injection of IP/placebo (Study Day 1), Surveillance (inpatient period Days 1–5), Day 15 Visit (Day 15 ± 2), and Day 29 Visit (Day 29 ± 3). The list of tests and procedures performed during the Visits is presented at Table 2.

**Table 2:** Tests and procedures performed during the study

Study stages	Screening	Injection of IP											Surveillance				Day 29 ± 3 End of study visit
Days of study	Day (-14) - 0	Day 1, hrs									Day 2, hrs		Day 3	Day 4	Day 5	Day 15 ± 2	
Hours after injection of Mobilan/placebo		Before injection	0	0.5	1	1.5	2	4	8	12	24	36					
Procedures		Inpatient period															
Obtaining written informed consent^1^	X																
Collection of past medical history data^2^	X																
The ECOG performance status	X																
Measuring height and weight	X																
Smoking status, cigarettes per day	X																
Calculating units of alcohol consumed per week	X																
Assessing patient’s eligibility for the clinical trial	X																
Thorough physical examination	X	X															X
Physical exam associated with patient’s complaints				X	X	X	X	X	X	X	X	X	X	X	X	X	X
Vital signs^3^	X	X		X	X	X	X	X	X	X	X	X	X	X	X	X	X
Breath alcohol and urine drug tests	X	X															
Complete blood count	X	X		X	X		X	X	X	X	X	X	X	X	X	X	
Serum chemistry profile	X	X									X		X	X	X	X	X
Coagulation profile	X	X								X			X	X	X	X	X
Clinical urine examination	X	X								X	X	X	X	X		X	
12-lead ECG	X	X						X		X	X	X	X	X	X		X
f/t PSA ratio test		X							X		X				X	X	X^4^
Cytokine assay		X					X	X	X	X	X	X	X	X	X		X
Immune cell count		X									X		X	X	X		
Analysis of 502s titer		X					X	X	X	X	X	X	X	X	X		X
PK_DNA_^5^		X		X	X		X	X				X					
Analysis of anti-502s antibodies		X															X
Injection of Mobilan/placebo into the prostate under control of ultrasonography			X														
Assessment of Adverse Events	Adverse Events were documented during the entire study starting immediately after signing the Informed Consent Form																

^1^Written informed consent must be obtained before initiating any study-related procedures.

^2^Including the serological tests for chronic infections, such as HIV, hepatitis B and C, syphilis, herpes, tuberculosis; collection of data on allergies and substance abuse; results of histological examination of prostate biopsy core; results of the urine drug and breath alcohol tests.

^3^Including blood pressure, respiratory rate, heart rate, body temperature, height, and weight.

^4^For the patients to undergo radical prostatectomy, blood samples for determining the f/t PSA ratio are collected preoperatively in the morning of RPE day.

^5^PK_DNA_ – pharmacokinetics of IP according to the level of Mobilan DNA vector in peripheral blood.

The decision as to whether the IP dose could be escalated in each subsequent cohort was made by the Expert Committee on Drug Safety (ECDS) based on the safety observations made in the previous cohort. The Study Protocol was approved by the Council of Ethics of the Ministry of Health and Local Ethical Committees of the participating clinical centers. Informed consent was obtained from all study subjects.

The treatment strategy for each patient (RPE or active surveillance) was chosen by the Principal Investigator (PI) in compliance with the routine clinical practice of the study center. Twenty-six patients in the study were assigned to operative treatment, with 20 scheduled to undergo RPE between Day 15 and Day 29 and 6 scheduled for RPE after Day 29. The remaining 6 patients were under active surveillance (no scheduled surgery). Patients scheduled by the PI to undergo RPE before Day 29 after injection of IP (*n* = 20) had their “Day 29” visit on the day of RPE (in the morning, prior to surgery). Such patients were then monitored until the period of 29 post-injection days was over, at which point the study was regarded as terminated for the patient. Safety data obtained after RPE were not included in the safety report. Surgical material obtained during RPE was forwarded to blinded histopathologist for histological analysis (see below). Patients scheduled by the PI to undergo radical prostatectomy after Day 29 post-injection (*n* = 6) and patients under surveillance (*n* = 6) completed their Day 29 visit as scheduled on Day 29, and those having RPE made an additional EoS Visit on the day of RPE prior to surgery. Surgical material obtained during RPE was forwarded for histological analysis. There were no cases of early withdrawal from the study.

It should be noted that since the primary purpose of this trial was safety assessment, a statistical plan was not made. Therefore, statistical analysis was not pre-defined and all calculations were made by research mood using Prism for OS X version 8.0.2.

### Safety

In this trial, safety was assessed through physical examinations including measurement of vital signs, evaluation of ECG data and laboratory values as mention in Table 2. The safety population in this study consisted of all patients who were given an injection of either investigational medicinal product or placebo. In accordance with the protocol, the safety data at Day 29/STV visit were excluded from analysis if the patient had undergone RPE at least 3 days earlier than Day 29 visit.

A total of 58 adverse events (AEs) were documented in 18 of the 24 patients treated with Mobilan (75%) and 11 AEs were documented in 4 of the 8 patients treated with placebo (50%) (Table 3). In the group of patients treated with Mobilan, 29 AEs were documented in 9 (100.0%) patients in cohort 1; 7 AEs, in 3 (100.0%) patients in cohort 2; 6 AEs, in 2 (66.7%) patients in cohort 3; 9 AEs, in 2 (66.7%) patients in cohort 4; and 7 AEs, in 2 (33.33%) patients in cohort 5. In the group receiving Mobilan (M-VM3), the intensity of all AEs according to CTCAE was grades 1, 2, and 3: 32 AEs in 14 (58.33%) patients were of grade 1; 18 AEs (including one SAE) in 11 (45.83%) patients were of grade 2, and 8 AEs (including 1 SAE) in 4 (16.67%) patients were of grade 3. In the placebo group, 7 AEs in 3 (37.5%) patients were of grade 1 and 4 AEs in 3 (37.5%) patients were of grade 2 according to the CTCAE classification. When classifying AEs according to their causal relationship to IP, the investigators qualified 48 AEs (in 18 (75%) patients treated with Mobilan) as drug-related (i. e., the causal relationship between these AEs and the IP could not be completely ruled out even if this causal relationship could not be proved or was unlikely) and 10 AEs (in 5 (20.83%) patients) as drug-unrelated. In the placebo group, all 11 reported AEs were classified as drug-unrelated.

**Table 3:** All adverse events (AEs) observed during the course of the study classified by system organ class and preferred term (number of patients, % of the total number of patients in the group)

Classes and preferred terms of AEs	Mobilan Cohort 1, *n* = 9	Mobilan Cohort 2, *n* = 3	Mobilan Cohort 3, *n* = 3	Mobilan Cohort 4, *n* = 3	Mobilan Cohort 5, *n* = 6	Placebo, *n* = 8
*Abnormal laboratory values*						
Prolonged thrombin time	1 (11.1%)					2 (25%)^*^
Elevated ESR	3 (33.3%)					
Elevated blood level of creatine phosphokinase	5 (44.4%)			4 (66.7%)^**^	2 (16.7%)	2 (25%)^*^
Elevated blood level of CPK-MB	2 (22.2%)					
Elevated C-reactive protein level	4 (44.4%)	2 (66.7%)	2 (66.7%)	1 (33.3%)	2 (33.3%)	
Elevated fibrinogen level	1 (11.1%)					
Increased neutrophil count	1 (11.1%)					
Elevated ALT level						1 (12.5%)
Elevated blood level of creatinine						1 (12.5%)
Prolonged aPTT						1 (12.5%)
Prolonged thrombin time						2 (25%)
*Disorders of the blood and hematopoietic system*						
Leukocytosis	3 (33.3%)	2 (66.7%)			1 (16.7%)	
Monocytosis	1 (11.1%)					
Thrombocytopenia			1 (33.3%)			
*Cardiovascular disorders*						
Hypertension	3 (11.1%)^*^					
Essential hypertension	1 (11.1%)					
Ventricular extrasystoles (quadrigeminy)	1 (11.1%)^*^					
*Renal and urinary disorders*						
Pollakiuria	1 (11.1%)		1 (33.3%)			
*Gastrointestinal disorders*						
Acute duodenal ulcer	1 (11.1%)^*^					
Acute gastric ulcer		1 (33.3%)^*^				
Ulcer in the lower thoracic esophagus			1 (33.3%)^*^			
Hyperbilirubinemia				1 (33.3%)	1 (16.7%)	
*Reproductive system disorders*						
Acute prostatitis		1 (33.3%)		1 (33.3%)		1 (12.5%)^*^
*Patient’s overall condition*						
Hyperthermia			1 (33.3%)	1 (33.3%)	1 (16.7%)	
*Metabolism and nutrition disorders*						
Hyperglycemia				1 (33.3%)^*^		2 (12.5%)^*^

The safety population in this study consisted of all patients who were given an injection of either investigational medicinal product or placebo. In accordance with the protocol, the safety data at Day 29/STV visit were excluded from analysis if the patient had undergone RPE at least 3 days earlier than Day 29 visit. ^*^ - not related to IP; ^**^ - possibly related to IP; absence of ^*^ - related to IP.

In the group treated with Mobilan (M-VM3), 32 AEs in 14 (58.33%) patients were classified by CTCAE as grade 1 AEs (13 AEs in 6 (66.7%) patients in cohort 1; 3 AEs in 2 (66.7%) patients in cohort 2; 5 AEs in 2 (66.7%) patients in cohort 3); 6 AEs in 2 (66.67%) patients in cohort 4; and 5 AEs in 2 (33.33%) patients in cohort 5); 18 AEs in 11 (45.83%) patients were classified as grade 2 AEs (9 AEs in 4 (44.44%) patients in cohort 1; 4 AEs (including 1 SAE) in 3 (100.0%) patients in cohort 2; 1 AE in 1 (33.3%) patient in cohort 3; 3 AEs in 2 (66.67%) patients in cohort 4; and 1 AE in 1 (16.67%) patient in cohort 5); and 8 AEs in 4 (16.67%) patients were classified as grade 3 AEs (7 AEs (including 1 SAE) in 3 (33.33%) patients in cohort 1 and 1 AE in 1 (16.67%) patient in cohort 5). Dose escalation did not increase the number of AEs of greater severity.

In the placebo group, 7 AEs in 3 (37.5%) patients were classified by CTCAE as grade 1 AEs and 4 AEs in 3 (37.5%) patients, as grade 2 AEs. Grade 3 AEs were not documented in the placebo group.

Forty-eight AEs in 18 patients treated with Mobilan (M-VM3) were qualified by the investigators as adverse drug reactions (ADRs); i. e., as being potentially related to IP: 24 AEs in 9 (100%) patients in cohort 1; 6 AEs in 3 (100.0%) patients in cohort 2; 5 AEs in 2 (66.7%) patients in cohort 3; 6 AEs in 2 (66.67%) patients in cohort 4; and 7 AEs in 2 (33.33%) patients in cohort 5. The number of AEs related to IP did not increase in dose cohorts after dose escalation.

The most frequent adverse events were abnormal laboratory values. Among patients treated with Mobilan, the most frequent AEs related to IP included: 5 cases of elevated creatine phosphokinase level in 4 (44.44%) patients and 4 cases of elevated C-reactive protein level in 4 (44.44%) patients in cohort 1; 2 cases of elevated C-reactive protein level in 2 (66.67%) patients in cohort 2; 2 cases of elevated C-reactive protein level in 2 (66.67%) patients in cohort 3; 4 cases of elevated creatine phosphokinase level in 2 (66.67%) patients and one case of elevated C-reactive protein level in one (33.33%) patient in cohort 4; 2 cases of elevated creatine phosphokinase level in one (16.67%) patient and 2 cases of elevated C-reactive protein level in 2 (33.33%) patients in cohort 5. Two cases of elevated creatine phosphokinase level were observed in 2 (25%) patients in the placebo group.

Among patients treated with Mobilan, the following adverse events qualified as disorders of blood and the hematopoietic system were documented: in cohort 1, 3 AEs in 3 (33.33%) patients (leukocytosis); in cohort 2, 2 AEs in 2 (66.67%) patients (leukocytosis); in cohort 3, one AE in one (33.33%) patient (thrombocytopenia); in cohort 4, 0 AEs; in cohort 5, one AE in one (16.67%) patient (leukocytosis). No adverse events classified as disorders of blood and the hematopoietic system were documented in the placebo group.

Among patients treated with Mobilan, the following adverse events qualified as renal and urinary disorders were documented: in cohort 1, one AE in one (11.11%) patient (pollakiuria); in cohort 2, 0 AEs; in cohort 3; one AE in one (11.11%) patient (pollakiuria); in cohort 4, 0 AEs; and in cohort 5, 0 AEs. No adverse events classified as renal and urinary disorders were documented in the placebo group.

Among patients treated with Mobilan, the following adverse events qualified as reproductive system disorders were documented: in cohort 1, 0 AEs; in cohort 2, one AE in one (33.33%) patient (acute prostatitis); in cohort 3, 0 AEs; in cohort 4, one AE in one (33.33%) patient (acute prostatitis); and in cohort 5, 0 AEs. One case of acute prostatitis in one (12.5%) patient was documented in the placebo group.

The following disorders of patient’s overall condition were documented: in cohort 1, 0 AEs; in cohort 2, 0 AEs; in cohort 3, one AE in one (33.33%) patient (hyperthermia); in cohort 4, one AE in one (33.33%) patient (hyperthermia); and in cohort 5, one AE in one (16.67%) patient (hyperthermia). No AEs classified as disorders of patient’s overall condition were documented in the placebo group.

Two SAEs were documented during the study: one in a Mobilan-treated patient in cohort 1 (severe pollakiuria with leukocytosis and elevated C-reactive protein level) and one in a Mobilan-treated patient in cohort 2 (acute prostatitis). These SAEs were classified as possibly related to administration of IP. Neither deaths nor patient withdrawal from the study because of drug-related AEs and SAEs occurred during the trial.

### Pharmacokinetics

Mobilan DNA was not detected by PCR in plasma prepared from peripheral blood samples collected from Mobilan-treated patients at any of the time points (Table 2). This indicates that, consistent with our animal studies [13], there was not significant leakage of Mobilan from the site of injection.

We also measured levels of 502s protein and anti-502s antibodies in blood plasma samples collected on Study Days 1–5 and EoS visit in order to confirm expression of 502s.

Immunological detection and quantification of 502s was performed using enzyme-linked immunosorbent assay (ELISA). The assay follows “sandwich” ELISA scheme where 502s is first bound by capture antibody and then detected by biotinylated primary detection antibody, which is in turn bound by streptavidin conjugated to enzyme producing fluorescent product upon addition of substrate. We did not detect 502s protein in the plasma of any Mobilan-treated patients at any of the tested post-injection time points (all obtained values were below the lower limit of detection of the assay).

**Figure 3:**
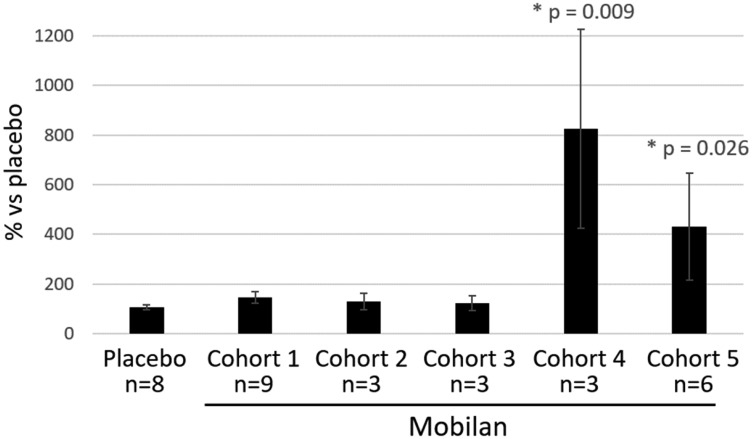
Titer of anti-502s antibodies in peripheral blood plasma of patients injected with Mobilan (M-VM3) or placebo. The anti-502s antibody titer on Day 29 after IP/placebo injection on Study Day 1 is shown normalized to the baseline titer measure on Day 1 before injection (set at 100%. Mean values ± SEM are shown. ^*^ *P* value < 0.05 (ordinary 1-way ANOVA test) shows comparison of normalized Mobilan value to normalized placebo value.

Anti-CBLB502 antibody titers in serum samples were determined using ELISA method in “bridging” format by coating plates with non-labeled protein drug (CBL502), incubating with diluted serum samples and detecting the bound anti-drug polyclonal antibodies with the labeled drug (Biotin-502). We did observe elevation of anti-502s antibody titers in the plasma of patients from the two cohorts that received the highest doses of Mobilan. As shown in Figure 3, mean anti-502s antibody levels at the Day 29 study visit were significantly higher than baseline in Mobilan-treated subjects of cohort 4 (3 × 10^10^ virus particles) and cohort 5 (1 × 10^11^ particles), but not in those treated with lower doses of Mobilan or with placebo. These results provide indirect verification of 502s protein production in Mobilan-injected patients.

### Pharmacodynamic effects of Mobilan on cytokine levels

Elevated plasma cytokine levels are a marker of inflammation [14, 15]. Pharmacological activation of TLR5 leads to induction of a number of cytokines, including, most prominently, IL-6, IL-8 and G-CSF [13]. These factors are likely key mediators of the immunoregulatory activity of TLR5 agonists [16]. Therefore, plasma concentrations of IL-6, IL-8 and G-CSF were analyzed as pharmacodynamic markers of Mobilan activity in this study using commercially available specific ELISA assays and peripheral blood samples collected from Mobilan-treated and placebo-treated patients at multiple time points over the first five days post-injection and at the EoS visit.

The cytokine assay involved evaluation of levels of interleukins IL-6, IL-8, and G-CSF. Levels of blood cytokines were measured at time points Day 1 (pre-injection), Day 1 (2 h), Day 1 (4 h), Day 1 (8 h), Day 1 (12 h), Day 2 (24 h), Day 2 (36 h), Day 3, Day 4, Day 5, Day 15, Day 29/EoS, and RPE day using enzyme-linked immunosorbent assay.

Sandwich enzyme-linked immunosorbent assay (ELISA) was carried out using 96-well microplates with pre-adsorbed mouse monoclonal anti-human IL-8/NAP-1 antibodies, mouse monoclonal anti-human IL-6 antibodies, or monoclonal anti-human G-CSF antibodies. Antibody-bound cytokines were detected using biotin-conjugated polyclonal anti-IL-8/NAP-1 antibodies, anti-IL6 mouse monoclonal antibodies or polyclonal anti-human C-GSF antibodies, respectively, and horseradish peroxidase-conjugated streptavidin. Tetramethylbenzidine solution was used as a substrate for horseradish peroxidase. The minimum detectable cytokine concentrations measured as the mean value + 2 standard deviations in 6 replicas for a 0 pg/ml (diluent solution) was 2.0 pg/ml for IL-8, 0.92 pg/ml for IL-6, and 11 pg/ml for G-CSF.

**Figure 4:**
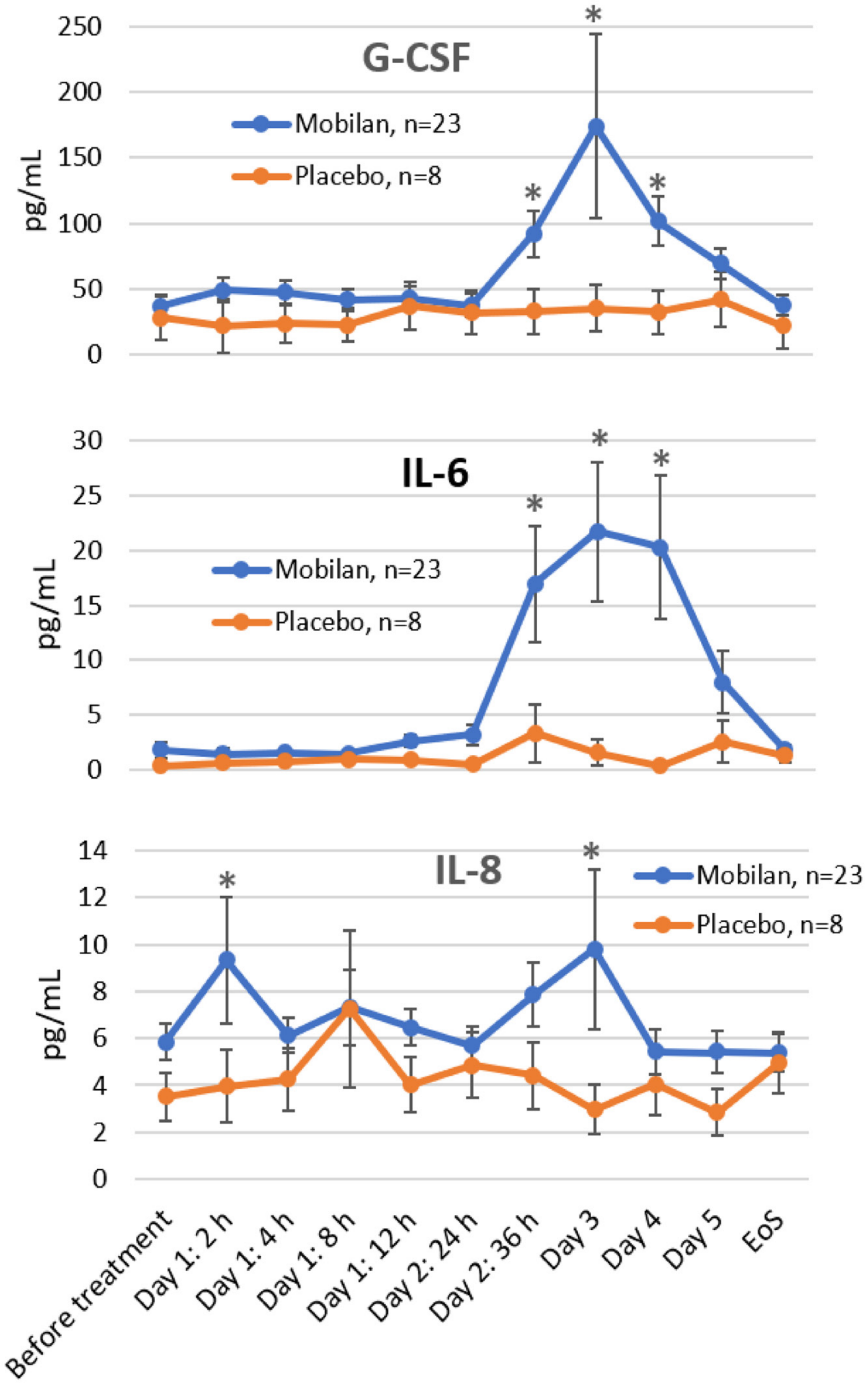
Effect of Mobilan injection on plasma levels of cytokines. G-CSF, IL-6 and IL-8 were measured in plasma samples collected from study subjects at the indicated times before or after Mobilan/placebo injection on Study Day 1 using specific ELISA assays. Mean values ± SEM for all cohorts combined are shown. *P* value < 0.05 (way ANOVA test). Extreme values (≥ 10-fold higher than the group mean) reflecting individual patient variability were excluded from the analysis. ^*^ *P* value < 0.05 (multiple *t*-test). EoS = end of study visit.

As shown in Figure 4, both G-CSF and IL-6 showed strong induction following Mobilan, but not placebo, administration, with peak levels observed on Study Day 3 (48 hours post-injection). Including all Mobilan-treated subjects, the maximum increases in mean G-CSF and IL-6 concentrations over the corresponding baseline values were 4,5-fold and 7-fold. Minor elevation (< 2-fold) of IL-8 was observed in Mobilan-treated patients at some time points, but the results were not statistically significant. The results obtained here for G-CSF and IL-6 clearly demonstrate the immunostimulatory efficacy of Mobilan administered to humans by intra-prostatic injection.

### Pharmacodynamic effects of Mobilan on PSA level

**Figure 5:**
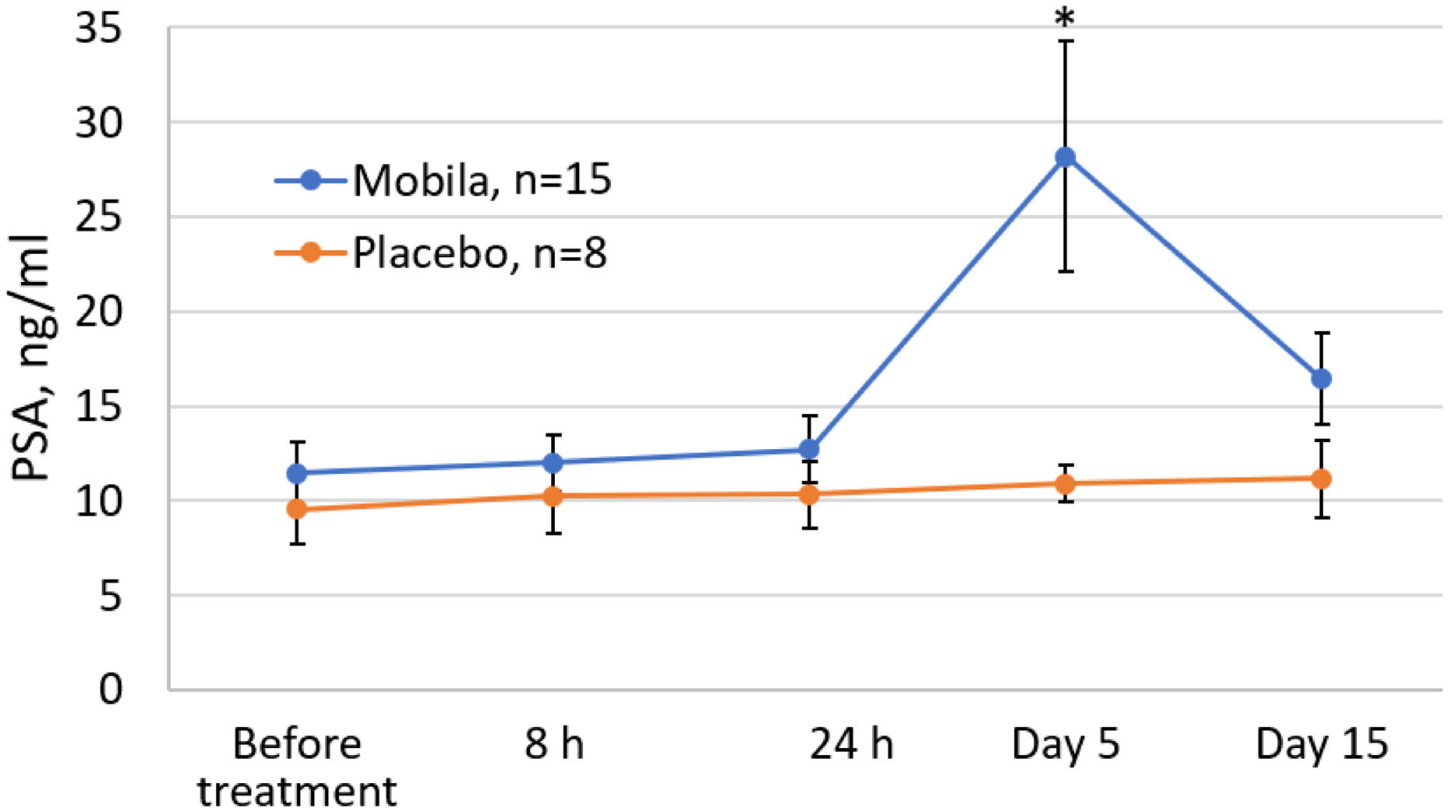
Effect of Mobilan injection on total PSA levels in the serum of prostate cancer patients (ng/mL). PSA levels were measured by chemilumescent assay in serum samples collected at the indicated timepoints relative to Mobilan or placebo injection on Study Day 1. Mean values ± SEM for all cohorts combined are shown. Day 29/EoS data are not shown because some study subjects underwent RPE before that visit (which had a significant effect on PSA levels), while other subjects did not have surgery before Day 29. No study subjects had RPE surgery before Day 15. ^*^ *P* value < 0.05.

In prostate cancer, PSA is easily detectable biomarker, which allows one to diagnose the disease and monitor its progression. To determine the effect of intra-prostatic injection of Mobilan on PSA levels in human PC patients, we used a chemiluminescent assay to analyze serum samples collected from study subjects before injection, at 8 and 24 hours post-injection, and at Day 5 and 15 post-injection. While there was not a significant increase in mean PSA level in the placebo group over this time frame, there was a significant increase in Mobilan-treated subjects on Study Day 5 (Figure 5). This result, indicative of Mobilan-induced inflammation in the prostate tissue of treated patients, provides further support for the therapeutic action of Mobilan as a stimulator of innate immunity in this clinical trial.

### Pharmacodynamic effects of mobilan on peripheral blood immune cell counts

Since the mechanism of action of Mobilan involves immunologic response, we compared counts of different types of immune cells in peripheral blood samples from Mobilan-treated and placebo-treated patients by flow cytometry. Changes were observed in Mobilan groups, but not the placebo group, for the following parameters: T-lymphocyte-to-WBC ratio, absolute count of CD3+CD4+ T helper cells, NK-cells CD3-CD (16+56)+, TNK-cells CD3+CD (16+56)+, total T cells (CD3), CD19+ B lymphocytes, and null lymphocytes. However, since most changes lay within the normal range, the lack of significant effect of Mobilan on immune cell count in peripheral blood can be due to its local effect.

### Histopathological evaluation of the effect of Mobilan on prostate tissue structure

As a preliminary evaluation of Mobilan efficacy in inducing antitumor immune responses in PC patients, prostate tissue samples collected from study subjects upon RPE were processed for H&E staining and analyzed by a blinded trained histopathologist. First, samples were assigned a Gleason score, used to determine the aggressiveness of prostate cancer, which also reflects the degree of tissue differentiation (range = 2–10, with higher scores indicating more poorly differentiated/more advanced disease. As summarized in Table 4, comparison of these post-treatment scores to those assigned to the patient prior to study initiation (indicated in medical histories), showed that there was a slight increase in mean Gleason score in both Mobilan-treated and placebo-treated subjects (0.41 points and 0.43 points, respectively). Thus, there was a small, and similar, decrease in the degree of prostate tissue differentiation during the course of this study for both IP-treated and control groups. Mean Gleason scores in the three lowest dose Mobilan cohorts (Cohorts 1–3) showed small increases similar to that seen for all Mobilan subjects. On the other hand, Cohort 4 showed a small decrease in mean Gleason score and Cohort 5 had the greatest change in mean Gleason score with a 1.00 increase. Due to the lack of substantial difference between Mobilan and placebo groups and the absence of any clear Mobilan dose-dependent effect on Gleason scores, it was not possible to draw any conclusions from this analysis.

**Table 4:** Gleason scores based on histopathological evaluation of prostate tissue structure before and after Mobilan (or placebo) treatment

		Mobilan (M-VM3)-treated cohorts					All Mobilan (M-VM3)-treated subjects	Placebo-treated subjects
		Cohort 1: 1 × 10^9^ particles	Cohort 2: 3 × 10^9^ particles	Cohort 3: 1 × 10^10^ particles	Cohort 4: 3 × 10^10^ particles	Cohort 5: 1 × 10^11^ particles		
Before injection of IP / placebo^1^	*n*	9	3	3	3	6	24	8
	Mean	6.44	6.33	6.33	6.33	6.00	6.29	6.17
	SD	0.53	0.58	0.58	1.15	1.26	0.81	0.75
After injection of IP / placebo^2^	*n*	6	3	3	2	6	20	5
	Mean	6.67	6.67	6.67	6.00	7.00	6.70	6.60
	SD	1.03	0.58	0.58	0.00	0.63	0.73	0.55

^1^Data from patients’ medical history (biopsy analysis), ^2^Data from assessment of prostate samples collected during RPE surgery; majority of patients had surgery between Day 15 and Day 29 after injection of IP/placebo on Study Day 1.

**Figure 6:**
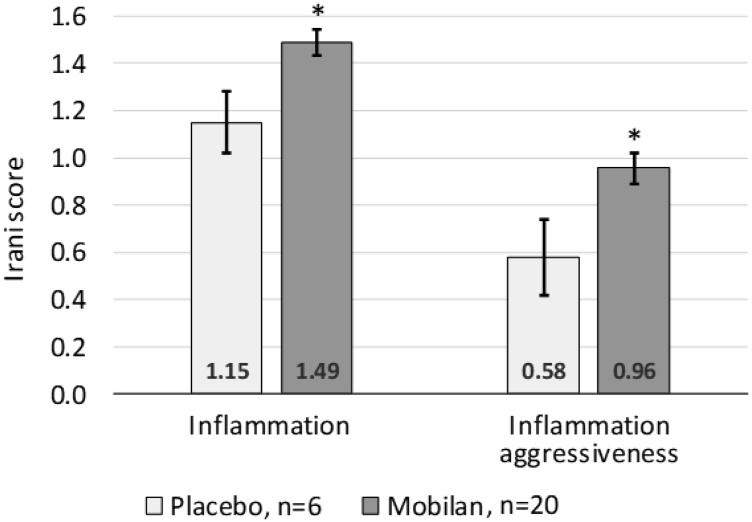
Irani scores for degree of lymphoid infiltration (left) and aggressiveness of lymphoid infiltration (right) assigned to prostate tissue samples collected during RPE from study subjects treated with Mobilan (M-VM3) or placebo. RPE took place after Day 15 according to IP prescription. Mean ± SEM for all cohorts combined is shown. ^*^ *P* value < 0.05 (multiple *t* test).

Histopathological assessment of prostate tissue sections collected in this study also included assignment of an Irani score, which provides a measure of the degree and aggressiveness of inflammatory lymphoid infiltration [17]. The scores shown in Table 5 indicate that all five cohorts of Mobilan-treated patients had a greater level of and more aggressive lymphoid infiltration than placebo-treated patients. A trend towards dose-dependence was observed in the Mobilan cohorts, with mean Irani scores for both degree of lymphoid infiltration and aggressiveness increasing with increasing Mobilan dose. The highest mean Irani score for the degree of lymphoid infiltration was observed in cohort 4 patients treated with Mobilan at a dose of 3 × 10^10^ particles and the highest score for lymphoid infiltration aggressiveness was observed in cohort 5 treated with Mobilan at a dose of 1 × 10^11^ particles. Figure 6 shows that the mean Irani scores for all five Mobilan cohorts combined were significantly higher than those for the placebo group. This indicates more profound inflammation in prostate tissues of Mobilan-treated patients versus controls, which is consistent with therapeutic efficacy of the drug.

**Table 5:** Irani scores indicating the degree of lymphoid infiltration and aggressiveness of lymphoid infiltration in prostate tissue samples collected from study subjects at the time of RPE, mean ± SD

Treatment group	Irani score	
	Degree of infiltration	Aggressiveness of infiltration
Mobilan (M-VM3), Cohort 1, 1 × 10^9^ particles, *n* = 6	1.42 ± 0.35	0.85 ± 0.40
Mobilan (M-VM3), Cohort 2, 3 × 10^9^ particles, *n* = 3	1.55 ± 0.15	0.86 ± 0.14
Mobilan (M-VM3), Cohort 3, 1 × 10^10^ particles, *n* = 3	1.47 ± 0.22	0.98 ± 0.36
Mobilan (M-VM3), Cohort 4, 3 × 10^10^ particles, *n* = 2	1.64 ± 0.04	1.06 ± 0.14
Mobilan (M-VM3), Cohort 5, 1 × 10^11^ particles, *n* = 6	1.50 ± 0.24	1.07 ± 0.27
Placebo, *n* = 6	1.15 ± 0.32	0.59 ± 0.38

## DISCUSSION

Mobilan (M–VM3) is an innovative gene therapy agent consisting of a recombinant non-replicating bicistronic adenovirus directing constitutive expression of TLR5 (from the CMV promoter) and the secreted flagellin-based TLR5 agonist, 502s (from the UbiC promoter). Such specific targeted vectors based on adenovirus (Ad) serotype 5 have emerged as commonly used and promising gene therapy agents [22]. Infection of tumor cells with Mobilan *in vivo* establishes local paracrine/autocrine TLR5 signaling leading to induction of antitumor innate, and subsequently, adaptive immune responses in the tumor microenvironment (Figure 1). Based on preclinical studies that provided proof of principle for this mechanism of tumor cell killing and identified prostate cancer (PC) as a potential target for Mobilan-based immunotherapy, this first-in-human Phase 1 trial was designed to evaluate the safety and tolerability, pharmacokinetics and pharmacodynamics of Mobilan in PC patients. The trial followed a study design that is widely used and considered standard for this type of trial. This design allows collection of the data of interest (including safety data) while minimizing risks for study subjects by including meticulous in-patient monitoring of the patient’s condition for 5 days post-injection, 4-week surveillance post-injection with a focus on organs/systems most sensitive to potential toxic effects of the drug, and decisions regarding dose escalation made by the Expert Committee on Drug Safety.

Mobilan was administered via intraprostatic injection in this study. This administration route was selected based on the results of a number of studies in which Mobilan was injected into various types of tumor tissue *in vivo* and expression of genes from the Mobilan construct was evaluated *ex vivo* [13, unpublished results]. The strongest expression was observed in prostate tumor samples. Moreover, intraprostatic injection of Mobilan in animal studies was shown to induce mobilization of immune cells at the injection site [13]. Therefore, given the available data, injection of Mobilan directly into the prostate of PC patients was expected to provide the best therapeutic effect. This strategy is also supported by the similar use of intraprostatic injection in PC patients followed by prostatectomy or active surveillance in a number of completed or ongoing clinical trials of new anticancer drugs. Overall, the intraprostatic administration route has been shown to be safe in clinical studies.

Due to the potential risks associated with the invasive intraprostatic administration route it was not feasible to enroll healthy volunteers in this phase I clinical trial. Therefore, the trial was designed for patients with a histologically-verified diagnosis of prostate cancer.

A minimal number of subjects (1 per cohort) receiving placebo treatment (5% glucose solution) was included in this trial to allow for proper interpretation of adverse events (AEs) related to intraprostatic injection. Placebo-treated control groups are often used in phase I studies in order to properly assess safety of the investigational product, including trials of adenovirus-based drugs [18, 19] and drugs administered intraprostatically [clinicaltrials. gov identifiers NCT00918983 and NCT00681148].

AEs that could possibly arise from intraprostatic injection itself (not related to Mobilan) include urethral and/or rectal bleeding, hematospermia, acute prostatitis, and delayed urination. Similar AEs could also occur during the harvesting of biopsy cores from the prostate [NCCN Clinical Practice Guidelines in OncologyTM Prostate Cancer Early Detection], which was conducted for all study subjects prior to their enrollment to confirm their PC diagnosis.

For this study, mobilan was injected bilaterally into both lobes of the prostate using ultrasound as a guide. Bilateral injection was selected over unilateral injection based on the assumption that it would provide more intense activation of the immune system and recruitment of immune cells into different regions of the prostate gland as foci of Mobilan-induced TLR5 signaling formed. Furthermore, sextant biopsy data currently do not allow unambiguous localization of prostate cancer, as indicated by the high percentage (72%) of false-positive diagnoses of unilateral cancer revealed by retrospective analysis [20]. In particular, a long-term clinical trial of medicinal product AdV-tk based on adenovirus vector containing the herpes simplex virus thymidine kinase gene has recently been completed and demonstrated that the medicinal product is safe and well tolerable when administered intraprostatically followed by prostatectomy [21]. It was thorough examination of cross-sections of the prostate tissue after RPE that allowed one to assess the pharmacokinetic parameters of vector distribution over the volume of the entire prostatic gland and local changes caused by administration of the medicinal product.

Since some cell types in mammals (macrophages, some populations of dendritic cells, and small intestinal epithelium) normally express TLR5 and TLR5 agonists are safe within a clinically determined permissible range, it is fair to suggest that safety will be evaluated for Mobilan injected into patients’ prostate. For example, the study to assess safety of a similar immunotherapeutic drug based on adenoviral vector for treating prostate cancer upon intraprostatic administration showed no significant drug-related adverse events or delayed toxicity during a long-term follow-up [21].

In this study, most of the observed AEs were abnormal laboratory values characterized as Grade 1 or Grade 2 according to the CTCAE classification system and were not accompanied by any clinical symptoms or patients’ complaints. Most of the clinically significant elevations in laboratory values appeared to result from the effect of the IP. Nevertheless, we conclude that Mobilan demonstrated satisfactory safety and tolerability at all doses analyzed in this study. The finding that all tested doses of Mobilan were safe indicates that the maximum tolerated dose (MTD) was not reached in this study. It should be noted that the highest Mobilan dose level tested here corresponds to the maximum concentration of the drug that can be obtained during its production.

The study also demonstrated that intraprostatic injection of Mobilan had the expected pharmacodynamic effects on a number of parameters confirming functionality of the construct and representing its known mechanism of action as an immunoregulatory agent. Thus, Mobilan (but not placebo) injection led to temporal elevation of total PSA levels and plasma cytokine (G-CSF, IL-6, IL-8) levels, elevation of anti-502s antibody titer, and an increased degree of lymphoid infiltration and aggressiveness of lymphoid infiltration in prostate tissue. Importantly, it was demonstrated previously that the presence of anti-502s antibodies, while confirming expression of 502s, does not interfere with the ability of Mobilan to activate TLR5 signaling in infected cells [13]. Mobilan-induced production of pro-inflammatory cytokines such as G-CSF and IL-6 provides a clear pharmacodynamic biomarker of the drug’s *in vivo* activity that is intimately connected to its mechanism of action. It should be noted that the lag observed between Mobilan administration and cytokine induction in this study (48 hours) was longer than that seen after injection of the TLR5 agonist entolimod [16]. It is likely that this time is needed to reach sufficient levels of expression of Mobilan-encoded TLR5 and 502s and allow for their interaction.

While there were several positive pharmacodynamic indications of Mobilan activity in the patients in this study, we did not observe a clear effect of the drug on tumor Gleason score which would suggest a therapeutic effect. Mobilan-treated and placebo-treated patients exhibited a similar decrease in the degree of cell differentiation estimated by the Gleason scoring system over the course of the study. However, since an effect of the IP on cell differentiation may take longer to develop than the time frame of this study, we were not able to draw any unambiguous conclusions from this analysis.

Also, it is fair to conclude that the optimal correlation between safety parameters and pharmacodynamic parameters (indicating that inflammatory response is stimulated, which is an anticipated and desired effect in accordance with the mechanism of action of IP) was observed for patients in cohort 5 treated with an injection of Mobilan at a dose of 1 × 10^11^ particles.

Therefore, the results of this first-in-human clinical trial provide strong support for further development of Mobilan as a PC treatment. This addresses the critical need for new anticancer drugs that persists despite our improved understanding of the mechanisms controlling development and progression of malignant neoplasms. New strategies are needed to overcome the issues of tumor cells’ acquisition of resistance to therapy and tolerance to the body’s immune system as well as the high toxicity of many existing anticancer drugs that limits their therapeutic effects within permissible dose ranges. Key advantages of Mobilan over existing PC solutions (e. g., hormone therapy and cytostatic systemic chemotherapy) are (i) local delivery via 1–2 injections vs. systemic treatment involving multiple doses, (ii) a favorable toxicity profile, and (iii) the possibility of targeting both hormone-dependent and hormone-resistant cancer. Moreover, the mechanism of action of Mobilan suggests that it may have synergistic effects when administered in conjunction with existing anticancer drugs, particularly immune checkpoint inhibitors such as PD-1 inhibitors (Opdivo, Keytruda) or CTLA-4 inhibitors (Yervoy). Further study of such combinations in preclinical models of PC or other cancers may lead to expansion of the potential therapeutic applications of Mobilan.

## MATERIALS AND METHODS

### Mobilan

The investigational product (IP) Mobilan (M–VM3) is a recombinant non-replicating bicistronic adenovirus that directs expression of the human Toll-like receptor 5 (hTLR5) protein and a specific TLR5 ligand, the flagellin derivative 502s [13].

Mobilan is produced as a concentrated stock solution (10^12^ viral particles/ml), which is then diluted to prepare working solutions for intratumor injection. The pilot batch of Mobilan for clinical trials was produced by IMMAPHARMA LLC (Russian Federation) in compliance with Good Manufacturing Practice (GMP) regulations. Mobilan particles were produced by homologous recombination using a human embryonic kidney cell culture transformed with the E1 region of human adenovirus serotype 5 (HEK293) followed by stepwise chromatographic purification. One ml of Mobilan stock solution contains the active pharmaceutical ingredient, 10^12^ physical MOBILAN viral particles (corresponding to 10^10^ plaque-forming units), and the following excipients: tris (hydroxymethyl) aminomethane, 2.424 mg; sodium chloride, 1.46 mg; glycerol, 0.025 mg; and water for injection, up to 1 ml. The number of replication-competent particles was meticulously verified during quality control and release testing for each batch of Mobilan. The Mobilan stock solution is stored at –70°C and thawed and diluted with 5% glucose solution to the required concentrations immediately before administration.

### Inclusion criteria

1. Written informed consent for participation
2. Males ≥ 45 and ≤ 75 years old
3. Patients with the histologically verified diagnosis of prostate cancer (stages T1-T2, N0, M0)
4. Patient's ECOG performance status 0–2
5. Negative serological tests for HIV, viral hepatitis B and C, and syphilis
6. The patient and his partner must agree to use barrier contraceptive methods for the duration of the study.

### Exclusion criteria

1. Failure to obtain informed consent
2. Clinical or radiographic signs of metastatic disease
3. Indications for hormone therapy of prostate cancer
4. Clinically relevant cardiovascular diseases:
  - Myocardial infarction within 6 months prior to screening
  - Unstable angina within 3 months prior to screening
  - Severe insufficient blood circulation (grade III)
  - Clinically relevant heart rhythm disorders
  - Hypotension (systolic blood pressure < 86 mm Hg) or bradycardia with HR < 50 bmp
  - Uncontrolled hypertension (systolic blood pressure > 170 mm Hg or diastolic blood pressure > 105 mm Hg)
5. Past history of clinically relevant central nervous system diseases by the time screening is performed.
6. Existing infection or other severe or systemic disease increasing the risk of therapy complications.
7. Past history of pituitary or adrenal insufficiency.
8. Other malignant tumors within the past 5 years.
9. Past history of other relevant concurrent diseases that, in Investigator’s opinion, can be aggravated during the study, including uncontrolled diabetes mellitus, rectal disorders, rectal fissures, hemorrhoids, rectal polyps, rectal strictures, and inflammation of the urogenital system: chronic prostatitis, cystitis, urethral catheter, and chronic retention of urine.
10. Positive allergic history, systemic allergic reactions, any alimentary allergy, intolerance, restrictions or special diets that, in Investigator’s opinion, may be a contraindication for participation of the subject in this study.
11. Administration of drugs having a pronounced effect on the immune system within 3 months prior to screening, long-term administration of disaggregants (warfarin, low-molecular-weight heparin, except for Thrombo ASS).
12. The patient is currently participating in other clinical trials or was administering IP within 30 days prior to screening, or had persistent adverse drug reactions from any IP.
13. Any clinically relevant abnormal patient’s condition and/or laboratory values not mentioned in the Protocol and revealed at screening and/or any reason that, in Investigator’s opinion, can impede patient’s participation in the trial.
14. Drug or alcohol abuse (at screening or past abuse), which makes the patient ineligible for trial participation in Investigator’s opinion; consumption of more than 5 units of alcohol per week (one unit of alcohol is the equivalent to: 1/2 l of beer, 200 ml of wine, or 50 ml of spirits) or previous alcoholism, drug addiction, drug abuse and/or past history of severe alcohol dependence or excessive use of drugs causing drug dependence within one year prior to screening visit.
15. Vaccination within 14 days prior to study initiation
16. Smoking more than 10 cigarettes per day
17. Inability to understand or follow study instructions
18. Patient is unavailable for surveillance within 29 days after IP administration or is unable to adhere to the study visit schedule.
19. Idiosyncratic reaction to the components of IP.

### Endpoints

Presence or absence of dose-limiting toxicity, frequency and intensity of adverse events (according to the CTCAE classification [23]), the number of early discontinuation cases due to IP-related AEs and SAEs, changes in routine laboratory examination values (complete blood count, serum chemistry profile, coagulation profile, and clinical urine examination) and cytokine levels (G-CSF, IL-6, IL-8), electrocardiogram tracing, blood pressure, heart rate, and respiration rate values and physical examination findings were considered as safety endpoints.

The level of Mobilan expression vector in patient’s peripheral blood determined by validated qPCR assay was selected as pharmacokynetics endpoint. Serial dilution of DNA of Mobilan with the known copy number was performed; a pair of primers for amplification and the optimal conditions ensuring the desired amplification efficiency and linearity within the range between 11 and 3E+06 copies were selected: the calculated amplification efficiency was 95% and the coefficient of determination was 0.997. Mobilan DNA was detected within the entire tested range, from 1E+09 to 3.8E+03 particles per extraction point (from 200 μl of blood); hence, the detection threshold was 3.8E+03 particles per extraction point from 200 μl of blood. The calculated amplification efficiency for the calibration curve of Mobilan isolated from donor’s blood was 100.0%; the coefficient of determination was 0.99. Parameters of the calibration curve are fitted by linear curve; therefore, the linear region of the method is between 3.8E+03 and 1E+09 particles per extraction point. The mean percentage of extraction of Mobilan DNA from blood was 27% (in order to evaluate the extraction degree, the same Mobilan serial dilutions were added to PBS instead of volunteer’s blood under the same conditions). The specificity of the method for quantifying Mobilan DNA in blood was confirmed in blood of 6 donors with no Mobilan added: in the control blood samples from all donors not treated with Mobilan, no Mobilan DNA was detected. No cross-contamination between the wells containing and not containing Mobilan was revealed when extracting Mobilan DNA. Furthermore, no bias in the level of Mobilan detected in blood of different donors, which could have been caused by different degrees of extraction of Mobilan DNA from blood of different donors and other similar reasons, was detected. The results were analyzed using Prism 5.02 software (https://www.graphpad.com/).

Pharmacodynamic parameters determined in the study included an assessment of the level of prostate-specific antigen, immune cell count in patient’s whole blood evaluated by flow cytometry, histopathological evaluation of changes in the structure of prostate tissue using the Gleason score and assessment of the degree of lymphoid infiltration and the aggressiveness of the infiltration using the Irani scale [17] (if prostatectomy was conducted during the study and the material is available for analysis), plasma level of 502s and titer of anti-502s antibodies (ELISA method) in peripheral blood.

## Abbreviations

AE: adverse event
ALT: alanine aminotransferase
aPTT: activated partial thromboplastin time
CMV: cytomegalovirus
CPK-MB: creatine phosphokinase myocardial band
ECDS: Expert Committee on Drug Safety
EoS: End of Study visit
ESR: erythrocyte sedimentation rate
IL: interleukin
IP: investigational product
G-CSF: granulocyte colony stimulating factor
PSA: prostate-specific antigen
RPE: radical prostatectomy
SAE: serious adverse event
TLR: Toll-like receptor
UNL: upper normal level
